# Three Cases of Tape-Worm, with Remarks

**Published:** 1852-11

**Authors:** 


*Three Cases of Tape-Worm, with Remarks.* By Henry S. Patterson, M. D., Professor of Materia Medica and Therapeutics in Pennsylvania Medical College.

Case I. *Successful use of Kwosso.*—Miss W., æt. twenty-two years, consulted me in March, 1851, with tape-worm. She had been in failing health for several months, with headache, languor, variable appetite, nausea, and abdominal pains, but had not sought medical advice. Having taken a dose of citrate of magnesia, she observed in her discharges something peculiar, which proved to be joints of tænia solium. As I was at that time expecting some kwosso from Europe, I postponed treatment until it should arrive. Early in April it came, and was administered, during my absence from the city, by my friend Dr. Gilbert. He gave the dose (ʒvj.) at once, the patient having fasted from the previous day. It excited some nausea but no vomiting. It was followed, in a few hours, by a dose of castor oil, which brought away a tape-worm several yards in length, but which unfortunately was not preserved for more minute examination. There can be no doubt, however, that the entire worm was expelled, as the patient rapidly convalesced, and has been in the enjoyment of uninterrupted health since that period.

Case II. *Failure of Kwosso. Successful use of Pumpkin Seeds.*—The subject of this case was for some time under my care, in consultation with my colleague, Dr. Darrach. I can aver that he was most thoroughly put through the entire routine of tape-worm remedies, before he left Philadelphia. He tells his own story so well, that I prefer to give the following extract from a letter announcing his restoration to health: "In the early part of January, 1836, I was rather suddenly attacked with what seemed to be an alarming diarrhœa, which continued for some weeks, resisting the usual remedies. My symptoms had been peculiar for some time previous to the attack. Indeed, I had all the prominent symptoms of tænia as laid down in the books, viz., dizziness; occasional false vision; variable appetite; pain in the lumbar region; pain in the knee-joint; swelling of the abdomen; hesitancy of speech; restlessness in time of sleep; unusual drowsiness during the day; varying strength, being sometimes quite strong and then again quite feeble. Somewhere about the middle of February of the same year, I discharged, at a morning stool, about nine yards of the tænia. From that time onward, for six years, I was more or less under medical treatment continuously. I took vast quantities of the spts. turpentine, (once or twice two ounces at the dose,) also the malefern, calomel and jalap, and Jayne's vermifuge; and was several times under homœopathic treatment. I took also iodide of potassium, iodide of iron, decoction of pomegranate, and the 'kousso.' I discharged large quantities of the worm, but no head could be perceived. When the kousso failed, I began to despair of being cured at all, but my sister, Mrs. ——, sent me, in December last, two numbers of the Boston Medical and Surgical Journal, containing two several accounts of the cure of tænia, by the use of pumpkin seeds. Having previously abstained from usual food for a day, on the 20th of January last, I took, at 8 o'clock in the morning, two ounces of the kernels of pumpkin seeds pulverized with two table-spoonfuls of white sugar, commingled with a half pint of boiling water, making a very pleasant drink for a fasting man. I kept my bed, drinking frequently of cold water, and at 9½ o'clock I took an ounce of castor oil. At 10½ I drank a cup of hot black tea, and, in about two minutes, discharged about eight yards of the tape-worm, *with the head.* O, how I wept for joy, that I was again a free man, after a servitude of six sad years to this awful complaint. Since then, I feel like a new being in a new world. My life had often been a weary burden, and yet I grew fleshy and looked healthful. For months in succession I had discharged the worm daily, in pieces of six to eighteen inches, and also in gourd-seed form. I suppose that, without any over-estimate, I discharged during the six years of my affliction, about *four hundred yards!* The remedy is very simple. Were I a practicing physician I would never administer the turpentine for tape-worm; I sometimes fear that I have experienced irretrievable harm to my kidneys from using it. There is virtue in pumpkin seeds, doctor, even if it be a *Yankee notion.*"

CASE III. *Successful use of Xanthoxylon Fraxineum.*—For the following curious case I am indebted to my friend Dr. Thomas J. Turner, of Port Richmond:

J. R., æt. 41 years, is a workman in a chemical laboratory. In December, 1847, whilst a private in the British army in Ireland, he first perceived that he was afflicted with tape-worm. He states that he passed fifteen or twenty joints at almost every stool for a time, and on several occasions as much as thirty feet at once. The surgeon of his regiment treated him with Ol. Terebinth. ℥j. every other day. He also took tin-powder, malefern root, and "every other article he ever heard of." He finally abandoned the hope of a radical cure. The symptom most prominent was a sense of gnawing and beating at the epigastrium in the morning. He was obliged to eat before rising, as he otherwise became faint, and "had all sorts of queer feelings." His appetite was insatiable. While at Port Richmond, in the autumn of last year, he was attacked with tertian intermittent fever, for which he was recommended to take an infusion of prickly ash bark in brandy, a popular domestic remedy. He digested an ounce of the bark in a pint of brandy, and drank the whole during the apyrexia. The result was a most copious diaphoresis, as usual, and also some purgation, bringing away the entire worm. He has remained perfectly well since.

I have recorded the above cases, because of the intrinsic interest attached to each, and more especially because they serve to teach us moderation in our laudations of special remedies for tape-worm. There are many substances which, if given at the right time and in the right way, will destroy and expel the tænia solium, but there is not one of them which will not frequently disappoint the practitioner. The subject of each of the foregoing cases is vehement in praise of his or her "cure," and yet the judicious practitioner will give either of them in his next case with an abundant reservation of doubt as to the result. Similar accounts of the accidental cure of tape-worm are by no means rare. We have recently had in the medical journals a history (from the Ann. Univers. of Turin,) of a case in which a shepherd, to whom valerian had been prescribed for a nervous affection, plucked and took by mistake, a quantity of Conium maculatum. He came very near losing his life, but fortunately recovered with the loss only of a tape-worm, which revealed the source of the nervous affection in question. The account is headed "Efficacy of Conium maculatum in Tape-worm." Is it not true, however, that any acro-narcotic poison, given so close to the verge of life-taking, may dislodge and destroy a parasite in the bowels? I should certainly refuse to admit the conium into the list of anthelmintics on this showing. The remedies used in our cases are fortunately without danger, and may be tried safely.

As to the kwosso, the excitement that attended its introduction, appears to have subsided already, and it is to be presumed that the greater part of the 1400 pounds which the nephew of M. Rochet d'Hericourt admitted to Dr. Pereira that that gentleman had in store at Paris, still remains on hand. The first notice of this article I have met with, is in the Annuaire of Bouchardat for 1841, on the authority of M. d'Abbadie, somewhat notorious in African affairs of late years. It was subsequently brought to England by Dr. C. T. Beke, and was the subject of a paper read by Dr. Hodgkin, at a meeting of the British Association at York. (See London Medical Times, October 26, 1844.) The Annuaire of Bouchardat for the same year, contains a notice of an attempt at its chemical analysis by Stanislaus Martin. One of the most satisfactory accounts of it is found in a communication from M. Schimpfer, Governor of Adoa, in the Gazette Med. de Strasbourg, (Bouchard. Ann. 1849, p. 254.) He informs us that "it never completely expels the worm, or at least that rarely happens." He also states that it is but one of the remedies relied upon in tape worm by the Abyssinians, and he enumerates eight others. Dr. Beke (to whom I am indebted for an interesting note on the subject,) informs me that the natives with whom he conversed, did not rate the kwosso above several of their other remedies. An Abyssinian servant, brought to London by Dr. B., was treated by Dr. Hodgkin for tape-worm, first with kwosso, and *afterward* with Ol. Terebinth. The impression of Dr. B. was that the latter was the more efficacious, though neither finally removed the worm, and the man returned to Africa showing occasional symptoms of its presence. Dr. B. states that the natives who use the article, are in the habit of taking a dose every two months. He is also my authority for the orthography kwosso, as best representing the Abyssinian pronunciation.

There can be very little doubt that the recent excitement about the kwosso was, in a great measure, the result of a commercial speculation. It has subsided suddenly, and I presume that the originators of the plan have been disappointed. It is already perceived that, while the kwosso is undoubtedly an active vermifuge, it is by no means so infallible as was represented. On the whole I consider it inferior to the Oleum Terebinthinæ, which is perfectly safe when judiciously given, notwithstanding the protest of my correspondent. I have given it constantly in ℥j. doses, or in doses of ℥ss. with an equal quantity of Oleum Ricini, and have never seen any ill effect whatever. Should the kwosso be afforded at a reasonable price, it will probably take its place among our established means of cure, but I have no expectation of its being employed at the rate of $5 for the dose of six drachms.—*Philadelphia Medical Examiner.*

